# SCAI/ACR/APMA/SCVS/SIR/SVM/SVS/VESS Position Statement on Competencies for Endovascular Specialists Providing CLTI Care

**DOI:** 10.1177/1358863X221095278

**Published:** 2022-04-25

**Authors:** Beau M. Hawkins, Jun Li, Luke R. Wilkins, Teresa L. Carman, Amy B. Reed, David G. Armstrong, Philip Goodney, Christopher J. White, Aaron Fischman, Marc L. Schermerhorn, Dmitriy N. Feldman, Sahil A. Parikh, Mehdi H. Shishehbor

**Affiliations:** a University of Oklahoma Health Sciences Center, Oklahoma City, Oklahoma; b University Hospitals Harrington Heart & Vascular Institute, Cleveland, Ohio; c University of Virginia Health System, Charlottesville, Virginia; d University of Minnesota, Minneapolis, Minnesota; e University of Southern California, Los Angeles, California; f The Dartmouth Institute, Lebanon, New Hampshire; g Ochsner Clinic Foundation, New Orleans, Louisiana; h Icahn School of Medicine at Mount Sinai, New York, New York; i Beth Israel Deaconess Medical Center, Boston, Massachusetts; j NewYork-Presbyterian/Weill Cornell Medical Center, New York, New York; k NewYork-Presbyterian/Columbia University Irving Medical Center, New York, New York

This statement was endorsed by the American College of Radiology (ACR), American Podiatric Medical Association (APMA), Society of Interventional Radiology (SIR), Society for Vascular Medicine (SVM), Society for Vascular Surgery (SVS), Society for Clinical Vascular Surgery (SCVS), and Vascular & Endovascular Surgery Society (VESS) in September 2021.

## Introduction

Chronic limb-threatening ischemia (CLTI) is the advanced stage of peripheral artery disease (PAD) characterized by rest pain or tissue loss. Up to 2 million individuals have this condition in the United States, and prevalence is anticipated to grow owing to aging of the population and increase in atherosclerotic risk factors such as diabetes and renal disease.^1^ In addition to the threat of limb dysfunction and amputation, patients with CLTI are at a high risk of cardio- and cerebrovascular morbidity and mortality, with risk that exceeds that of most other cardiovascular patients. Within 1 year, 1 in 5 CLTI patients dies, and an additional one quarter will require major limb amputation.^2^

Care of the CLTI patient is complex, multifaceted, and multidisciplinary. Medical therapy, wound care, interpretation of noninvasive and invasive vascular testing, and the performance of revascularization procedures are integral to achieve limb salvage. Both surgical and endovascular revascularization have been established as effective treatment modalities that alleviate symptoms and promote healing. Decisions regarding revascularization strategy for individual patients are nuanced and depend in part on comorbidities, anatomy, functional status, conduit availability, presence of suitable bypass target, and other factors. Endovascular revascularization is performed by physicians across a variety of disciplines including vascular surgeons—the only specialty providing both endovascular and open surgical intervention—interventional radiologists, interventional cardiologists, and others.^3^ Irrespective of specialty, the endovascular specialist focused on CLTI should understand the role of surgical revascularization, understand the likelihood of short-term and long-term success with each type of revascularization, possess competencies that extend beyond catheter-based therapies, and integrate other CLTI team members into patient care to optimize chances of successful outcomes.

Opportunities to improve CLTI care are readily available on many fronts. Failure to prescribe optimal medical therapy to mitigate cardiovascular risk, limited use of smoking cessation programs, and the underutilization of revascularization procedures to prevent limb loss are examples where undertreatment may increase the risk of poor outcomes. However, revascularization failure and the misinterpretation of noninvasive vascular testing to identify macrovascular PAD may also represent scenarios where suboptimal care has been delivered. Evidence from published literature support the existence of these realities in modern CLTI practice.^4,5^ Moreover, amputation rates are disproportionately worse in blacks and other minorities and individuals of low socioeconomic status.^6,7^ To date, few initiatives have been successful in eradicating these CLTI care disparities.

One mechanism to improve outcomes in individuals with any disease state is to improve the competency of providers delivering that care. This concept is particularly relevant in CLTI, where much of the care is delivered by physicians in different clinical settings with varied skillsets and unique training experiences. While global guidelines exist surrounding care of the CLTI patient,^8^ to date, a single CLTI-specific competency document has not been developed.^9,10^ This multispecialty societal writing group convened to develop a position statement outlining competencies for endovascular specialists providing CLTI care. Through dissemination and use by clinicians, training programs, and professional societies focused on CLTI, this effort may ultimately enhance the outcomes of this population in need. Although equally important, this document does not address the competencies necessary for optimal vascular surgical care of the patient with CLTI.

## Document development methodology

This document has been developed according to the Society of Cardiovascular Angiography and Interventions (SCAI) Publications Committee policies for writing group composition, disclosure and management of relationships with industry (RWI), internal and external review, and organizational approval.

Following proposal submission and approval by the SCAI Publications Committee, professional societies with interest in CLTI care were invited to participate in document development. Each society was asked to nominate one representative to participate in the writing group. Final selections for the writing group were made by the chair and co-chairs (BMH, MS) and the writing group was approved by the SCAI Publications Committee. Ultimately, a diverse and experienced group of content experts was formed with representation from the following societies: American College of Radiology (ACR), American Podiatric Medical Association (APMA), Society for Cardiovascular Angiography and Interventions (SCAI), Society of Interventional Radiology (SIR), Society for Vascular Medicine (SVM), Society for Vascular Surgery (SVS), Society for Clinical Vascular Surgery (SCVS), and Vascular & Endovascular Surgery Society (VESS).

The writing group has been organized to ensure diversity of perspectives and demographics, multi-stakeholder representation, and appropriate balance of RWI. Relevant author disclosures are included in Appendix 1. Before appointment, members of the writing group were asked to disclose all financial relationships from the 12 months prior to their nomination. Most of the writing group disclosed no relevant financial relationships. Disclosures were periodically reviewed during document development and updated as needed. SCAI policy requires that writing group members with a current financial interest are recused from participating in associated discussions or voting on relevant recommendations. The work of the writing committee was supported exclusively by SCAI, a nonprofit medical specialty society, without commercial support. Writing group members contributed to this effort on a volunteer basis and did not receive payment from SCAI.

Members of the writing group participated in a series of conference calls, jointly developed competencies utilizing the Accreditation Council for Graduate Medical Education (ACGME) core competencies framework,^11^ and drafted the final manuscript. All recommended competencies are supported by a short summary of the evidence or specific rationale.

The draft manuscript was posted for public comment for 30 days in January 2021 and the document was revised to address pertinent feedback. The writing group unanimously approved the final version of the document. SCAI, ACR, APMA, SCVS, SIR, SVM, SVS, and VESS endorsed the document as official society guidance in September 2021.

## Unique aspects of CLTI care

Care of the CLTI patient is multifaceted, and decidedly more complex and unique compared to individuals with milder forms of PAD and those with other forms of cardiovascular disease. In addition to the well-established risk of cardio- and cerebrovascular morbidity and mortality with CLTI, one glaring distinction relates to the threat of limb loss. Major amputation is a devastating and life-altering event for many patients, and its prevention necessitates coordinated and thorough multidisciplinary care, prescription of optimal medical therapy, treatment of concomitant comorbidities, and prompt revascularization. Unfortunately, many patients do not receive this, and multiple studies have demonstrated that amputations continue to regularly occur without appropriate vascular assessment and revascularization procedures.^12^

The burden of cardiovascular comorbidities in the CLTI population is well documented. CLTI patients are often elderly and frail, features which increase risks associated with revascularization procedures.^13^ This is highlighted by higher complication rates with surgical bypass compared to endovascular intervention. No randomized trial has shown a survival advantage for endovascular compared to surgical revascularization in CLTI, and a post-hoc analysis suggested an advantage with surgical revascularization in the BASIL trial for those patients who survive >2 years.^14^ In addition to standard atherosclerotic risk factors like smoking, hypertension, and hyperlipidemia, both diabetes and chronic kidney disease are particularly potent risk factors. Population studies suggest that approximately one half of patients with CLTI have diabetes or end-stage renal disease.^15,16^ Moreover, symptomatic atherosclerotic disease in other vascular beds is common, with a significant proportion of CLTI patients having had prior acute coronary syndromes and cerebrovascular events.^15^ Recent observational studies have suggested that the burden of these comorbidities in the CLTI population is increasing.^17^

From an anatomical standpoint, both the severity and distribution of PAD is more complex in those with CLTI compared to that encountered in those with claudication. A retrospective analysis of 450 CLTI patients presenting for revascularization found that multilevel disease (aorto-iliac, femoropopliteal, or below-knee) was present in roughly two thirds, with lengthy occlusive tibial disease being the most commonly encountered lesion phenotype.^18^ The presence of complex tibial disease was even more apparent when examining the cohorts with diabetes and end-stage renal disease. Moreover, infra-mallelolar disease, while known to be a marker of adverse wound healing,^19^ is a prevalent finding in CLTI limbs. Preliminary data suggest that pedal angioplasty hastens short-term wound healing, but it remains uncertain if this translates into improvements in limb salvage.^20^ Additionally, vessel calcification is common in CLTI patients.^19^ This anatomic milieu is difficult from an endovascular standpoint and presents extreme technical challenges, often necessitating multilevel procedures (in single or staged fashion), occasionally niche devices to cross and treat complex lesions, and alternate access sites to reach distal lesions or cross chronic total occlusions. Given the complexity and multi-level nature of atherosclerotic disease burden, endovascular therapies in CLTI patients have higher technical failure and complication rates, along with reduced durability compared to similar approaches in the patient with lifestyle-limiting claudication.^21-23^ Accordingly, many patients with severe multilevel disease and CLTI may be better suited for bypass, in particular when there is tissue loss and the need for patency durable enough for wound healing, which often takes >6 months.^8^

Noninvasive vascular testing is essential in patients with CLTI. Physiologic testing, which includes entities such as the ankle-brachial index (ABI), toe pressures and toe-brachial index (TBI), Doppler waveforms, pulse volume recordings, photoplethysmography, and other perfusion parameters, is paramount in localizing disease, quantifying severity, and assessing for the presence of other pathology beyond macrovascular PAD that may contribute to limb symptoms. Such testing is also useful in quantifying the effects of revascularization, and for surveillance monitoring during short- and long-term follow-up. It is increasingly acknowledged, however, that many of these tests have limitations and are best used in combination with clinical assessment and other objective data to properly manage patients with CLTI. As an example, in a large cohort of more than 10,000 patients receiving revascularization procedures for CLTI, the ABI was normal in 24%, likely owing to vessel calcification from diabetes and renal dysfunction.^24^ This emphasizes the importance in CLTI of obtaining and interpreting additional objective perfusion measures such as toe pressures or TBI.

Imaging is the other category of noninvasive testing that is frequently used to guide patient management and includes computed tomography angiography (CTA), magnetic resonance angiography (MRA), and duplex ultrasonography (DUS). These studies help localize disease and assist with procedural planning. Importantly, each has limitations, and none may supplant the need for invasive angiography in certain CLTI patients, particularly when infrapopliteal and more distal disease is present. As an example, CTA is less accurate in characterizing tibial disease, particularly in calcified vessels, relative to other imaging modalities.^25^ Newer techniques such as time-resolved MRA and dual energy CTA can help with disease characterization in these cases.^26,27^

Wound assessment is an integral component of CLTI management. Not all wounds or limb symptoms are attributable to macrovascular PAD. Clinicians evaluating patients with wounds, particularly when revascularization is being considered, must be able to differentiate those of ischemic etiologies from other causes, and be able to initiate the appropriate diagnostic workup and evaluation when non-ischemic lesions are encountered. Basic tenets of wound and podiatric care, as part of a comprehensive CLTI management program, are essential for endovascular specialists before and after revascularization procedures.

In summary, revascularization is an important component of CLTI care, but successful patient outcomes are contingent upon the timely and appropriate delivery of numerous other therapies. For endovascular specialists regularly treating CLTI, competency in these unique aspects of CLTI care is needed to eradicate under-treatment and misdiagnosis, avoid preventable amputation, and improve cardiovascular outcomes in this population.

## Individual competencies

The training pathways and mechanisms of competency acquisition for CLTI care will vary between different specialties. Nonetheless, there are common skillsets that all endovascular specialists should possess to facilitate successful outcomes in CLTI patients. Table 1 lists these skillsets and should serve as a framework for the development of tools to assist endovascular proceduralists in assessing and improving competencies. These skills are organized according to the 6 general core competencies used by the ACGME and endorsed by most medical specialty boards.^11^ These competency domains are: medical knowledge, patient care and procedural skills, systems-based practice, practice-based learning and improvement, professionalism, and interpersonal and communication skills.

**Table 1.** Competencies for endovascular specialists.

**Medical knowledge**
Know peripheral arterial anatomy
Know the causes, epidemiology, and natural history of CLTI
Know the indications for noninvasive testing for patients with suspected or established CLTI
Know the indications for medical therapy and risk factor modification for CLTI
Know the indications and contraindications for peripheral angiography
Know the indications and contraindications for endovascular and surgical revascularization in CLTI
Know the risks and benefits of CLTI revascularization strategies, both endovascular and surgical, and how to tailor each based on patient preference, comorbidities, and anatomy
Know the endovascular technologies and techniques available to treat CLTI
Know the complications of CLTI revascularization procedures
Know the differentiating characteristics between arterial, venous, neurotrophic and atypical lower extremity ulcers
Know the basic management of non-CLTI wounds including ancillary testing and referral when appropriate
Know the aspects of podiatric care relevant to patients with CLTI
Know the principles of radiation safety
**Patient care and procedural skills**
Perform a focused history and physical examination in patients with CLTI
Interpret noninvasive vascular imaging, physiologic and perfusion testing in patients with CLTI, before and after revascularization procedures
Prescribe medical therapy before and after revascularization to mitigate cardiovascular risk and optimize limb outcomes
Select revascularization strategies that are patient-centric and guideline-based, utilizing other specialists where appropriate
Perform preoperative risk assessment for patients prior to vascular surgery
Evaluate and manage lower extremity wounds, including referring for ancillary testing and specialty care when appropriate
Evaluate and manage uncommon vascular disorders and those that may mimic CLTI
Perform endovascular revascularization in the aorto-iliac, femoropopliteal, and tibial territories
Select and perform alternate access
Manage complications related to CLTI procedures
Utilize limb surveillance testing after revascularization
**Systems-based practice**
Utilize an interdisciplinary and coordinated approach for CLTI patient management
Utilize cost-awareness and risk-benefit analysis in patient care
**Practice-based learning and improvement**
Identify and act on performance gaps identified through review of scientific studies, registries, and guidelines
Participate in quality improvement initiatives
Participate in scientific endeavors aimed at improving CLTI care
**Interpersonal and communication skills**
Communicate with and educate patients and families across a broad range of socioeconomic, ethnic, and cultural backgrounds
Communicate and work effectively with various professionals on the CLTI team
**Professionalism**
Practice within the scope of expertise and technical skills
Know and promote adherence to guidelines and appropriate use criteria.
Interact respectfully and with integrity with patients, families, and all members of the CLTI team

CLTI, chronic limb-threatening ischemia.

It is recognized that a spectrum of skillsets exists across many competencies. To account for this range in complexity, examples of competencies were created and stratified into “fundamental” and “advanced” categories and are listed in Table 2. For example, in the case of tibial endovascular revascularization, angioplasty of a tibial artery stenosis is relatively simple in contrast to the treatment of a lengthy calcified tibial chronic total occlusion, where more advanced techniques may be needed. Likewise, the prescription of an antiplatelet and high-potency statin is basic care that should be offered to all patients with PAD, but the initiation of a low-dose direct-acting oral anticoagulant (DOAC) to a CLTI patient to reduce risk of limb events following revascularization could be considered more complex. Note that this framework, in its current iteration, should not be used to restrict the clinical practice of operators not meeting “advanced” criteria, nor should it be used by healthcare systems to compare operators within the same specialty or across differing ones. Indeed, many clinical scenarios exist where advanced skillsets may neither be available nor necessary in order to properly care for a CLTI patient. Rather, this schema identifies the requisite skillsets that all endovascular specialists should possess to provide CLTI care and outlines higher-level competencies that are obtainable and advantageous as they may be impactful in terms of improving outcomes in a greater number of patients with CLTI.

**Table 2.** Select examples of advanced and fundamental skillsets for CLTI care.

Competency Domain	Skillset	Fundamental	Advanced
**Medical knowledge**	Anatomy Noninvasive testing	Know basic aortoiliac, femoropopliteal, and tibial anatomy Know indications for and types of LE arterial testing	Know tibial variants, know pedal loop anatomy Know novel imaging and perfusion modalities
	Medical therapy	Know basic medical therapies for PAD	Know emerging medical therapies with limb efficacy (eg PCSK9s, DOACs)
	Wounds	Differentiate basic wound types	Know the management of non-arterial wounds
**Patient care**	Noninvasive testing	Obtain arterial physiologic testing to quantify and localize PAD	Interpret venous insufficiency testing to guide management of mixed wounds
**Systems-based practice**	Interdisciplinary care	Discuss angiogram with surgeon to select revascularization modality	Develop weekly multidisciplinary limb conference to guide patient revascularization management
**Practice–based learning and improvement**	Quality improvement	Review complications at regular intervals	Participate in a longitudinal CLTI registry to benchmark results regionally and nationally

CLTI, chronic limb-threatening ischemia; CTO, chronic total occlusion; DOAC, direct-acting oral anticoagulant; PAD, peripheral artery disease; PCSK9, proprotein convertase subtilisin/kexin type 9; SFA, superficial femoral artery; TASC, Trans-Atlantic Inter-Society Consensus.

The medical knowledge competencies were developed to highlight the critical knowledge base required for treatment of CLTI. The parameters for defining clinical success are different when comparing patients presenting with claudication versus those presenting with CLTI. While these distinct clinical presentations may be viewed as a continuum along the disease process of PAD, the overall goals in CLTI are distinct. Moreover, differences exist in the prevalence, presentation, and treatment outcomes of CLTI based on sex, race, and socioeconomic status, and should be recognized by endovascular specialists.^28,29^

It is imperative that endovascular specialists have a fund of knowledge that incorporates the competencies as outlined in Table 1. At a fundamental level, the proceduralists should be able to interpret a lower extremity arteriogram and use that anatomic information to develop a revascularization strategy. Angiosome-based revascularization, while conceptually important, does have limitations that should be understood when formulating revascularization plans.^30^ At a more advanced level, experience and familiarity with pedal arch anatomy will aid in cases of complex CLTI.^31^ In addition, the knowledge base also includes clinical skills such as the clinical evaluation of patients with CLTI and the differentiation between lower extremity wounds.^32^ At a fundamental level, the proceduralist would be able to describe the physical exam and noninvasive test findings that may be used to differentiate between lower extremity ulcers.^33^ At a more advanced level, the proceduralist would identify lower extremity ulcers with a mixed etiology and determine optimal treatment strategies subsuming appropriate diabetic/neurotrophic and venous treatment. Endovascular specialists should have an understanding of the use of radiation producing equipment and appropriate management of operator, staff and patient dose reduction.^34^

There are a number of procedural competencies that are necessary for treating patients with CLTI. Endovascular specialists should understand the indications for and be able to perform revascularization across the aorto-iliac, femoropopliteal, and tibial segments. Related to this, use of limb stratification schemes such as the Wound Infection Ischemia (WIfI) and Global Vascular Guidelines’ Global Limb Anatomic Staging System (GLASS) classifications are important in determining the relative benefit of performing revascularization to promote limb salvage in patients with CLTI.^8,35^ These operators should be facile with the use of specialty devices and niche endovascular technologies to facilitate technical procedural success and to optimize long-term patency rates. The ability to perform endovascular revascularization through alternate access sites (e.g., pedal, distal superficial femoral, and radial arteries) is becoming an increasingly important skill to tackle complex lesion subsets. Not all operators will have all of the advanced procedural skillsets necessary to treat the most complex CLTI anatomy. In these instances, collaboration with or referral to more experienced CLTI revascularization specialists may optimize the chances of successful limb salvage. Indeed, many endovascular techniques such as deep venous arterialization and pedal loop reconstruction are evolving and may occupy an important role in the care of CLTI patients moving forward.^36,37^

While the endovascular component of procedural competencies is important, so too is knowledge of hybrid or surgical options, thereby underscoring the critical need for multidisciplinary care of the CLTI patient in order to achieve limb salvage. Additionally, it is crucial to have an understanding of the likelihood of successful restoration of pulsatile flow to the forefoot for wound healing with either an endovascular or surgical revascularization, as well as an understanding of the potential implications of a failed initial intervention.^5,38^ While not all endovascular specialists will have a surgical background, they should understand the basics of preoperative risk assessment, as well as clinical and anatomic characteristics that influence the selection of revascularization modalities.^39,40^ In many scenarios, endovascular, surgical, and hybrid revascularization may be options for individual patients. The specialist should understand the relative benefits and risks of these modalities and work in concert within a team that offers a surgical perspective to formulate best treatment plans. In this regard, endovascular specialists should understand the assessment of surgical bypass targets, how conduit availability may impact the durability and quality of surgical revascularization, and how to preserve potential anastomotic bypass sites when proceeding with endovascular techniques.

It is of vital importance that the endovascular proceduralist recognizes the multidisciplinary nature of care provided to the CLTI patient. The significance of collaborative and multidisciplinary care to achieve optimal patient outcomes has been discussed throughout the literature and cannot be overstated.^41,42^ At a fundamental level, this involves working closely with members of other specialties to ensure optimal medical, surgical, vascular, and wound care. Depending on the knowledge base, clinical expertise, and technical skillset of the endovascular specialist, this may involve collaboration in several aspects of CLTI care. In addition, the proceduralist should acknowledge clinical scenarios in which patient care would be improved with referral to a more advanced center or to a provider capable of providing further technical expertise. Multidisciplinary care within CLTI can be viewed as a continuum and, at an advanced level, it is expected that the proceduralist would work to develop, promote, and advance guidelines and recommendations regarding appropriate treatment algorithms for CLTI in a multidisciplinary fashion seeking input from medical and surgical specialists with shared patient care interests.

## Volume and experience in endovascular training

The writing committee believes that technical proficiency for endovascular operators is improved by procedural volumes and experience. However, given limited data quality, heterogenous effect sizes, and differential and evolving findings, the writing committee also believes there is an absence of evidence to clearly define a procedural volume threshold whereby competence in endovascular interventions for CLTI is manifest. As such, the group has elected not to recommend a requisite minimal procedural volume at this time.

Published training statements from a variety of specialty societies have suggested that physicians perform a minimum of 100 diagnostic peripheral angiograms in order to display competence.^9,10,43,44^ There is less consistency in recommended interventional procedure volumes, but most societies recommend a minimum of 50 to 80 peripheral interventions, the majority of which should be arterial in nature. None of the recommendations address endovascular interventions for CLTI specifically, nor do they attempt to account for the varying degrees of complexity inherent to lower extremity arterial interventions based on lesion phenotype (e.g., stenosis versus calcified chronic total occlusion), segment (e.g., aorto-iliac versus tibial), and patient characteristics.

Better evidence to help formulate training guidelines and allow a systematic approach to endovascular competency will be a key multispecialty priority in coming years. For example, training programs could have their trainees log CLTI procedures, stratified by segment and complexity, and submit these data to a central repository to accurately quantify the number and types of procedures that endovascular trainees are performing in CLTI patients during their training programs. Similar processes, though not specific to CLTI, already exist for some procedural specialties. One could envision such an endeavor being a collaborative effort amongst medical organizations who support the educational endeavors of endovascular specialists.

National CLTI registries may also prove beneficial. While existing registries such as the Society of Vascular Surgery Vascular Quality Initiative (SVS VQI) collect procedural and outcome data on many CLTI patients, the ability to account for trainee involvement in procedures is currently limited. Modifications to data collection instruments that incorporate trainee participation could afford opportunities to generate volume thresholds for endovascular CLTI specialists.

## Institutional requirements

Traditional training statements discuss institutional requirements and resources necessary for learners to obtain the requisite skillsets to become competent in their specialty of interest. Care of the CLTI patient involves multiple environments including urgent care facilities, outpatient longitudinal clinics, inpatient wards, and procedural areas such as office-based laboratories, angiography suites, and operating rooms. Additionally, areas providing ancillary services like vascular laboratories and wound care centers contribute significantly to the overall management of this population. As such, it may be best to conceptualize the environment of developing endovascular specialists as a CLTI care system rather than an institution. In this sense, the care system functions as a comprehensive habitat where all aspects of CLTI care can be offered to optimize chances of best patient outcomes. This concept has been previously described and emphasizes the multidisciplinary care mandated for this unique population.^45^

Elements of the CLTI care system that should be available to support competency acquisition include outpatient clinics, diagnostic testing facilities (e.g., accredited noninvasive vascular laboratory), and procedural areas. In regard to clinics, many CLTI patients need urgent evaluations for wounds, infections, ischemic rest pain, and cardiovascular comorbidities. As such, clinic infrastructure should be able to accommodate CLTI patients quickly and efficiently, avoiding unnecessary delays that may jeopardize patient care. Collaboration with podiatry and wound care centers is of paramount importance, and institutions should have established relationships to these services to facilitate timely evaluation and management of CLTI patients before and after revascularization. Many patients may lack the necessary resources or social support to undergo the in-person clinical evaluations. In these scenarios, use of telemedicine service may be a useful mechanism to combat these barriers to care.^46^

Noninvasive vascular testing is of obvious importance in CLTI. Substantial variation in pre-procedural testing occurs in patients with CLTI based on patient characteristics, resource availability, and operator biases. At a minimum, the ability to obtain imaging with either CT, MR, or DUS should be available, though many patients may not be candidates for contrast-based studies due to the presence of renal dysfunction. A high-quality, Intersocietal Accreditation Commission-accredited vascular laboratory is necessary to perform arterial physiologic testing, perfusion assessment, and associated venous studies that may be necessary in CLTI patients. In particular, acknowledging the limitations of the ABI in CLTI, objective markers of wound healing such as toe pressures and TCPO2 are valuable in the care of individual patients and may facilitate more rapid and efficient treatment decisions. The laboratory should offer comprehensive vascular testing to facilitate the acquisition of the Registered Physician in Vascular Interpretation credential (RPVI) for learners in these respective programs.^9^

Procedural areas should be equipped with imaging systems capable of performing high-quality digital subtraction angiography (DSA). Supporting technologies (e.g., ultrasound guidance) should be available to assist with standard arterial and alternate access. Endovascular interventions will span from the aorta to the distal tibial and pedal circulations. As such, the procedural laboratories should have a full complement of wires, catheters, and balloons compatible with 0.014”, 0.018”, and 0.035” systems. Niche devices including re-entry catheters, crossing devices, cutting or scoring balloons, and atherectomy devices should be available since they may be needed to treat the complex disease subsets encountered in CLTI. Intravascular imaging (e.g., IVUS) has been associated with improved limb salvage rates,^47^ and may be helpful in optimizing technical outcomes. The procedural area should be equipped with devices to manage emergent complications, and if the facility is not within a hospital setting, systems should be in place to rapidly triage and transfer patients to acute care facilities when such complications arise.

## Competency acquisition

There are several avenues for acquiring the necessary clinical, didactic, and hands-on training for CLTI. The intensity of training and clinical exposure varies based on the pathway chosen: formal training or independent courses in a post-training practice.

### Formal training programs

Post-graduate, traditional training programs can take form in one of three different training tracks: vascular surgery (VS), interventional cardiology (IC), or interventional radiology (IR). Aside from the hands-on procedural training for CLTI, residents and fellows also undergo clinical training focusing on patient management, wound care, and certification for vascular interpretation as a part of these programs.

Vascular surgery training can be obtained in either a traditional vascular fellowship (5+2) program or an integrated vascular residency (0+5) program. In the traditional program, trainees undergo general surgery training for 5 years, followed by a 2-year sub-specialty fellowship training in vascular surgery. The integrated program, approved in 2006, allows a more focused sub-specialty training for a longer period. Both training paradigms have yielded positive training experiences and desired practice placement.^48^

Interventional cardiologists complete internal medicine residency and general cardiology fellowship, which are 3 years each in duration.^49^ Interventional cardiology fellowship has traditionally been a 1-year training experience with emphasis on coronary intervention. Many 1-year programs do offer peripheral training as well, and depending on the program, some do offer exposure to opportunities to acquire additional skillsets such as the RPVI certification. Moreover, vascular medicine is a requisite component of general cardiology fellowship, and most interventional cardiology fellows will have completed multiple months of vascular medicine rotations prior to beginning procedural fellowships. Given the complexity of CLTI, interventional cardiology fellows who plan to focus on CLTI should strongly consider pursuing advanced endovascular training, such as an additional year of peripheral vascular fellowship.

With the advent of advanced endovascular, structural heart, and increasingly complex coronary interventions, 2-year interventional cardiology or advanced endovascular fellowships are now becoming common in many academic centers. Many of these advanced programs allow for the acquisition of non-procedural skillsets and fulfill criteria to become board-eligible in vascular medicine.^9^ The American Board of Vascular Medicine currently offers board certification in general vascular medicine as well as endovascular medicine (available at vascularboard.org). The requirements for eligibility vary somewhat based on training program but include a minimum of 100 and 50 diagnostic and interventional procedures for endovascular certification, respectively, and a minimum of 12 months in a training program that offers comprehensive rotations in noninvasive vascular medicine for general certification.

Interventional radiology training is currently available via three routes. All trainees start with 1 year of a clinical internship. Pathways thereafter diverge and can include one of the following: (1) 3 years of diagnostic radiology with 3 months of interventional radiology, followed by 2 years of dedicated interventional radiology training (integrated IR residency); (2) 4 years of diagnostic radiology, which includes at least 3 months of interventional radiology, followed by 2 years of dedicated interventional radiology training (independent IR residency); (3) 4 years of diagnostic radiology with 12 months of interventional radiology and 500 image-guided procedures, followed by 1 year of interventional radiology training (early specialization).

The selected training path across each of these disciplines will depend upon individual trainee goals and career trajectory, as all of these specialties have non-endovascular components as well. Specifically, a vascular surgery practice will have a component of open surgery, an interventional cardiology pathway will incorporate coronary interventions and potentially structural heart interventions, and an interventional radiology track will also include diagnostic film interpretation and non-vascular interventions.

Regardless of the discipline and pathway, developing endovascular specialists to focus on CLTI is likely best achieved in a training environment that offers interdisciplinary team-based care, appreciates the modern role of surgical treatment (revascularization and limb salvage procedures), and emphasizes the importance of non-procedural skillsets such as vascular imaging and medical care. Many fellowships may benefit from collaborating across specialties to ensure trainees are allowed adequate exposure to these skillsets that are outside of their primary disciplines.

### Post-training courses

For those already in clinical practice, there are industry-sponsored opportunities to travel to high-volume centers for endovascular courses. These programs are typically composed of one to two days of intensive cases to allow demonstration in various aspects of endovascular procedures. Topics may include alternative access, ultrasound-guided access, crossing techniques, calcium modification, drug delivery, and device-specific usage. Simulations, proctored cases, and “double-scrubbing” with experienced operators are additional ways for established practitioners to obtain hands-on experience.

Compared to formal training, post-training independent learning has the advantages of exposure to endovascular practice variability throughout the country and being able to “learn on the job” without interruption of one’s established clinical practice. Disadvantages include a relatively minimal hands-on experience compared to the full immersion offered by traditional training pathways, lack of formal guidance on long-term CLTI patient clinical management before and after endovascular procedures, absence of standardization of training techniques, and significant risk of device-specific bias.

### Lifelong learning

As with all other facets of medicine, the technologies and techniques utilized in the endovascular space will continue to evolve as new discoveries arise to help optimize wound healing in the CLTI population. The key for long-term success is engagement in lifelong learning through local, national, and international conferences, to continue to share ideas across the wide spectrum of endovascular practices, and to stay up to date on advancements in care in this complex patient population. Given the importance of technological innovation in endovascular therapies, industry-supported training programs will remain an important source of education for CLTI operators. Educational organizations are uniquely positioned to develop CLTI-specific continuing medical education (CME) content that providers may access to enhance performance.

## Future directions

In the future, there are multiple mechanisms by which these competencies may be utilized to improve endovascular specialists’ care in CLTI. At a training program level, this framework may allow program directors and faculty to develop curricula or rotations targeting specific educational gaps. While these needs may be fulfilled within the same specialty, some skillsets may be best acquired through education by specialists in different disciplines, as often CLTI experts arise from a variety of disciplines within single institutions. Such interdisciplinary collaboration is attractive in CLTI given the unique perspectives and skillsets that clinicians across a variety of discipline can provide.

Much emphasis has been appropriately centered around a “CLTI team” care model for this population. Wide variation exists at an individual and institutional level regarding team components and the services rendered by individuals within the care team. Institutions training residents, fellows, or practicing physicians to specialize in CLTI may use this competency-based framework to develop comprehensive programs fulfilling these educational needs. Ultimately, while immature at present, a CLTI certification process may be helpful to objectively appraise individual and institutional performance.

## Conclusions

The common goals of all specialties engaged in the care of CLTI patients are to optimize quality of life, reduce cardiovascular morbidity and mortality, and eradicate preventable amputation. CLTI will continue to be managed by multiple specialists from diverse training backgrounds, and as such, standardizing expected competencies for endovascular specialists is a necessary step to ensure that patient-centric and evidence-based therapy is delivered. The framework presented in this document is a starting point to enable training programs, professional medical societies, and other entities to develop curricula to optimize skillsets for clinicians focusing on this clinical niche. Ultimately, through the identification of common needs spanning across multiple endovascular specialties, such an effort may spark collaborative inter-disciplinary education efforts, and ultimately enhance the care that this population so desperately needs.

## Supplemental Material

sj-pdf-1-vmj-10.1177_1358863X221095278 – Supplemental material for SCAI/ACR/APMA/SCVS/SIR/SVM/SVS/VESS Position Statement on Competencies for Endovascular Specialists Providing CLTI Care Click here for additional data file. Supplemental material, sj-pdf-1-vmj-10.1177_1358863X221095278 for SCAI/ACR/APMA/SCVS/SIR/SVM/SVS/VESS Position Statement on Competencies for Endovascular Specialists Providing CLTI Care by Beau M. Hawkins, Jun Li, Luke R. Wilkins, Teresa L. Carman, Amy B. Reed, David G. Armstrong, Philip Goodney, Christopher J. White, Aaron Fischman, Marc L. Schermerhorn, Dmitriy N. Feldman, Sahil A. Parikh and Mehdi H. Shishehbor in Vascular Medicine
