# Supplementary material for: SCAI/ACR/APMA/SCVS/SIR/SVM/SVS/VESS Position Statement on Competencies for Endovascular Specialists Providing CLTI Care
Source: Vasc Med. 2022 Apr 25;27(4):405–14. doi: 10.1177/1358863X221095278 (PMC9344564; doi:10.1177/1358863X221095278)
Supplement: sj-pdf-1-vmj-10.1177_1358863X221095278 – Supplemental material for SCAI/ACR/APMA/SCVS/SIR/SVM/SVS/VESS Position Statement on Competencies for Endovascular Specialists Providing CLTI Care [file sj-pdf-1-vmj-10.1177_1358863X221095278.pdf]

Writing Group Disclosure Summary

Per SCAI policy, all nominees to this writing group were asked to complete a Disclosure of Relationships with Industry Form prior to their appointment.

Among this group, 0 out of 14, or 0%, have current, relevant relationships with industry that may represent actual or perceived conflicts of interest. The Chair and Vice-Chair disclosed no such relationships with industry.

Group Member	Description of Relevant Relationship(s)	Management Strategy and Rationale
Beau Hawkins (Chair)	No relevant relationships.	This group member is part of the unconflicted majority.
Mehdi Shishehbor (Vice-Chair)	No relevant relationships.	This group member is part of the unconflicted majority.
David G. Armstrong	No relevant relationships.	This group member is part of the unconflicted majority.
Herb Aronow	No relevant relationships.	This group member is part of the unconflicted majority.
Teresa Carman	No relevant relationships.	This group member is part of the unconflicted majority.
Dmitriy Feldman	No relevant relationships.	This group member is part of the unconflicted majority.
Aaron Fischman	No relevant relationships.	This group member is part of the unconflicted majority.
Philip Goodney	No relevant relationships.	This group member is part of the unconflicted majority.
Jun Li	No relevant relationships.	This group member is part of the unconflicted majority.
Sahil A. Parikh	No relevant relationships.	This group member is part of the unconflicted majority.
Amy B Reed	No relevant relationships.	This group member is part of the unconflicted majority.
Marc Schermerhorn	No relevant relationships.	This group member is part of the unconflicted majority.
Christopher J White	No relevant relationships.	This group member is part of the unconflicted majority.
Luke Wilkins	No relevant relationships.	This group member is part of the unconflicted majority.
